# Tris(thio­cyanato-κ*N*)tris­(triphenyl­phosphine oxide-κ*O*)europium(III)–(nitrato-κ^2^ *O*,*O*′)bis­(thio­cyanato-κ*N*)tris­(triphenyl­phosphine oxide-κ*O*)europium(III) (1/1)

**DOI:** 10.1107/S1600536812047472

**Published:** 2012-11-24

**Authors:** Anthony T. Thames, Frankie D. White, Lam N. Pham, Kang Rui Xiang, Richard E. Sykora

**Affiliations:** a University of South Alabama, Department of Chemistry, Mobile, AL 36688, USA

## Abstract

The title co-crystal, [Eu(NCS)_3_(C_18_H_15_OP)_3_][Eu(NCS)_2_(NO_3_)(C_18_H_15_OP)_3_], contains two distinct neutral complexes. Each complex has threefold symmetry about its central Eu^3+^ ion. As a result, the nitrate-containing mol­ecule contains disorder of its bidentate nitrate and two *N*-bound thio­cyanate anions, while the [Eu(NCS)_3_(OPPh_3_)_3_] complex is fully ordered. There is a weak π–π stacking inter­action between the phenyl rings of the two mol­ecules [centroid–centroid distance = 4.138 (4) Å].

## Related literature

For structural studies on related *f*-block triphenyl­phosphine oxide complexes, see: Feazell *et al.* (2004 ▶); Berthet *et al.* (2003 ▶); Long *et al.* (1999 ▶); Bowden *et al.* (2010 ▶). For syntheses and spectroscopic characterization of related compounds, see: Cousins & Hart (1967 ▶, 1968 ▶).
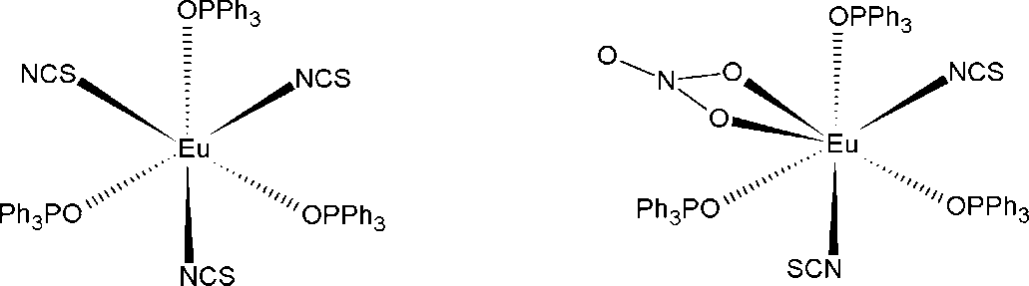


## Experimental

### 

#### Crystal data

[Eu(NCS)_3_(C_18_H_15_OP)_3_][Eu(NCS)_2_(NO_3_)(C_18_H_15_OP)_3_]
*M* *_r_* = 2325.95
Trigonal, 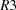

*a* = 20.3249 (7) Å
*c* = 22.3186 (6) Å
*V* = 7984.7 (4) Å^3^
*Z* = 3
Mo *K*α radiation
μ = 1.42 mm^−1^
*T* = 180 K
0.12 × 0.07 × 0.05 mm

#### Data collection

Agilent Xcalibur Eos diffractometer
Absorption correction: multi-scan (*CrysAlis PRO*; Agilent, 2012 ▶) *T*_min_ = 0.892, *T*_max_ = 1.000
15970 measured reflections
6325 independent reflections
5614 reflections with *I* > 2σ(*I*)
*R*_int_ = 0.052

#### Refinement

*R*[*F* ^2^ > 2σ(*F* ^2^)] = 0.042
*wR*(*F* ^2^) = 0.065
*S* = 1.02
6325 reflections
439 parameters
8 restraints
H-atom parameters constrained
Δρ_max_ = 1.30 e Å^−3^
Δρ_min_ = −0.90 e Å^−3^
Absolute structure: Flack (1983 ▶), 3154 Friedel pairs
Flack parameter: −0.045 (9)

### 

Data collection: *CrysAlis PRO* (Agilent, 2012 ▶); cell refinement: *CrysAlis PRO*; data reduction: *CrysAlis PRO*; program(s) used to solve structure: *SHELXS97* (Sheldrick, 2008 ▶); program(s) used to refine structure: *SHELXL97* (Sheldrick, 2008 ▶); molecular graphics: *OLEX2* (Dolomanov *et al.*, 2009 ▶); software used to prepare material for publication: *publCIF* (Westrip, 2010 ▶).

## Supplementary Material

Crystal structure: contains datablock(s) I, global. DOI: 10.1107/S1600536812047472/qm2089sup1.cif

Structure factors: contains datablock(s) I. DOI: 10.1107/S1600536812047472/qm2089Isup2.hkl

Additional supplementary materials:  crystallographic information; 3D view; checkCIF report

## Figures and Tables

**Fig. 1.**
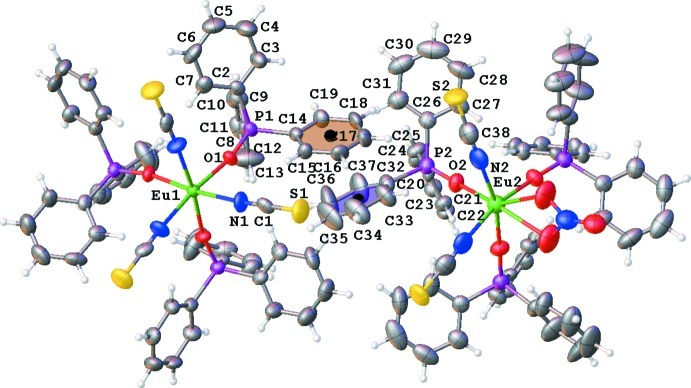
The molecular structure of I, with the atom-numbering scheme. Displacement ellipsoids for non-hydrogen atoms are drawn at the 50% probability level.

## supplementary crystallographic information

### Comment

From previous studies, it has been known that lanthanide triphenylphosphine oxide complexes can be prepared with a number of anions including nitrate (Cousins & Hart, 1967; Long *et al.*, 1999), thiocyanate (Cousins & Hart, 1968; Feazell *et al.*, 2004), bromide (Bowden *et al.*, 2010), trifluoromethanesulfonate (Berthet *et al.*, 2003), and iodide (Berthet *et al.*, 2003). The title compound, [Eu(OPPh_3_)_3_(SCN)_3_][Eu(OPPh_3_)_3_(SCN)_2_NO_3_], is of particular interest because of the anion disorder in one of the two neutral molecules in this co-crystal. There are two crystallographically unique europium(III) sites in the structure, one at the center of each neutral complex, [Eu(OPPh_3_)_3_(SCN)_3_] and [Eu(OPPh_3_)_3_(SCN)_2_NO_3_], as shown in Fig. 1. Each complex has threefold symmetry and therefore the two thiocyanato and one bidentate nitrate anions are disordered over the three positions in the latter. In [Eu(OPPh_3_)_3_(SCN)_3_], the three triphenylphosphine oxide ligands and three thiocyanto anions are found in a *fac* arrangement. The isolation of the mixed-anion system is interesting from a coordinating viewpoint, as the thiocyanato displays a monodentate (*kN*) coordination while the nitrato is bidentate (*κ^2^O,O*'). The thiocyanato-*kN* coordination is also observed in previous lanthanide complexes, such as [Nd(OPPh_3_)_4_(SCN)_3_] (Feazell *et al.*, 2004), likely due to the hard nature of the Ln(III) ions. Also of note is the fact that the title compound contains a 1:3 ratio of Eu(III) to phosphine oxide in both of its complexes, whereas with the larger Nd(III) ion, four triphenylphosphine ligands coordinate. Regarding intermolecular interactions, the title compound contains one weak π-stacking interaction, with plane-to-centroid distances of 3.529 (8) and 3.841 (5) Å, between adjacent rings of the two complexes as illustrated in Fig. 1.

It should be noted that the stoichiometry of the reaction conditions to prepare the title compound were not rigorously controlled and it is likely that the introduced nitrate is a result of a slightly less than 1:3 ratio of Eu(III) to KSCN.

### Experimental

Ethanol solutions of europium(III) nitrate hydrate (~1 mmol) and KSCN (~3 mmol) were combined. The resultant solution was decanted from the KNO_3_ precipitate. This solution was then mixed with an ethanol solution of triphenylphosphine oxide (~4 mmol). Within one hour, the colorless crystals suitable for the X-ray analysis were isolated.

### Refinement

H-atoms were placed in calculated positions and allowed to ride during subsequent refinement, with *U*_iso_(H) = 1.2*U*_eq_(C) and C—H distances of 0.95 Å.

### Crystal data

**Table:**

[Eu(NCS)_3_(C_18_H_15_OP)_3_][Eu(NCS)_2_(NO_3_)(C_18_H_15_OP)_3_]	*D*_x_ = 1.451 Mg m^−^^3^
*M**_r_* = 2325.95	Mo *K*α radiation, λ = 0.71073 Å
Trigonal, *R*3	Cell parameters from 5442 reflections
*a* = 20.3249 (7) Å	θ = 3.2–25.0°
*c* = 22.3186 (6) Å	µ = 1.42 mm^−^^1^
*V* = 7984.7 (4) Å^3^	*T* = 180 K
*Z* = 3	Prism, colourless
*F*(000) = 3534	0.12 × 0.07 × 0.05 mm

### Data collection

**Table:**

Agilent Xcalibur Eos diffractometer	6325 independent reflections
Radiation source: Enhance (Mo) X-ray Source	5614 reflections with *I* > 2σ(*I*)
Graphite monochromator	*R*_int_ = 0.052
ω scans	θ_max_ = 25.1°, θ_min_ = 3.2°
Absorption correction: multi-scan (*CrysAlis PRO*; Agilent, 2012)	*h* = −24→23
*T*_min_ = 0.892, *T*_max_ = 1.000	*k* = −22→24
15970 measured reflections	*l* = −26→26

### Refinement

**Table:**

Refinement on *F*^2^	Secondary atom site location: difference Fourier map
Least-squares matrix: full	Hydrogen site location: inferred from neighbouring sites
*R*[*F*^2^ > 2σ(*F*^2^)] = 0.042	H-atom parameters constrained
*wR*(*F*^2^) = 0.065	*w* = 1/[σ^2^(*F*_o_^2^) + (0.0112*P*)^2^] where *P* = (*F*_o_^2^ + 2*F*_c_^2^)/3
*S* = 1.02	(Δ/σ)_max_ = 0.001
6325 reflections	Δρ_max_ = 1.30 e Å^−^^3^
439 parameters	Δρ_min_ = −0.90 e Å^−^^3^
8 restraints	Absolute structure: Flack (1983), 3154 Friedel pairs
Primary atom site location: structure-invariant direct methods	Flack parameter: −0.045 (9)

### Special details

**Table:**

Geometry. All e.s.d.'s (except the e.s.d. in the dihedral angle between two l.s. planes) are estimated using the full covariance matrix. The cell e.s.d.'s are taken into account individually in the estimation of e.s.d.'s in distances, angles and torsion angles; correlations between e.s.d.'s in cell parameters are only used when they are defined by crystal symmetry. An approximate (isotropic) treatment of cell e.s.d.'s is used for estimating e.s.d.'s involving l.s. planes.
Refinement. Refinement of *F*^2^ against ALL reflections. The weighted *R*-factor *wR* and goodness of fit *S* are based on *F*^2^, conventional *R*-factors *R* are based on *F*, with *F* set to zero for negative *F*^2^. The threshold expression of *F*^2^ > 2σ(*F*^2^) is used only for calculating *R*-factors(gt) *etc*. and is not relevant to the choice of reflections for refinement. *R*-factors based on *F*^2^ are statistically about twice as large as those based on *F*, and *R*- factors based on ALL data will be even larger.

### Fractional atomic coordinates and isotropic or equivalent isotropic displacement parameters (Å^2^)

**Table:**

	*x*	*y*	*z*	*U*_iso_*/*U*_eq_	Occ. (<1)
Eu1	0.6667	0.3333	0.305072 (13)	0.02675 (13)	
Eu2	0.0000	0.0000	0.462118 (16)	0.04265 (17)	
P2	0.16058 (9)	0.14280 (10)	0.55807 (6)	0.0392 (4)	
C20	0.1572 (3)	0.1238 (3)	0.6370 (2)	0.0325 (13)	
C26	0.1639 (3)	0.2324 (3)	0.5478 (2)	0.0422 (15)	
C27	0.0969 (4)	0.2320 (4)	0.5345 (2)	0.0556 (18)	
H27	0.0511	0.1848	0.5302	0.067*	
C25	0.1888 (3)	0.1809 (3)	0.6797 (2)	0.0433 (15)	
H25	0.2129	0.2327	0.6680	0.052*	
C23	0.1489 (3)	0.0872 (3)	0.7568 (2)	0.0476 (16)	
H23	0.1458	0.0748	0.7981	0.057*	
C21	0.1214 (3)	0.0488 (3)	0.6551 (2)	0.0416 (15)	
H21	0.0993	0.0096	0.6259	0.050*	
C5	0.6419 (4)	0.6382 (4)	0.3299 (3)	0.063 (2)	
H5	0.6622	0.6894	0.3173	0.075*	
C7	0.6563 (3)	0.5304 (3)	0.3457 (2)	0.0447 (15)	
H7	0.6869	0.5072	0.3435	0.054*	
C22	0.1170 (3)	0.0297 (3)	0.7152 (2)	0.0484 (16)	
H22	0.0926	−0.0220	0.7274	0.058*	
C24	0.1848 (3)	0.1616 (3)	0.7400 (2)	0.0538 (17)	
H24	0.2071	0.2004	0.7694	0.065*	
C3	0.5402 (3)	0.5249 (4)	0.3722 (2)	0.0508 (16)	
H3	0.4900	0.4981	0.3879	0.061*	
C31	0.2310 (4)	0.3011 (4)	0.5537 (2)	0.0543 (18)	
H31	0.2775	0.3025	0.5619	0.065*	
C4	0.5701 (4)	0.5993 (4)	0.3524 (3)	0.069 (2)	
H4	0.5402	0.6232	0.3545	0.083*	
C32	0.2453 (3)	0.1481 (3)	0.5283 (3)	0.0455 (15)	
C28	0.0957 (6)	0.2984 (5)	0.5274 (3)	0.076 (3)	
H28	0.0494	0.2968	0.5175	0.091*	
C6	0.6848 (4)	0.6037 (4)	0.3256 (2)	0.0566 (19)	
H6	0.7343	0.6304	0.3088	0.068*	
C15	0.4249 (3)	0.2996 (3)	0.3298 (2)	0.0411 (14)	
H15	0.4606	0.2973	0.3037	0.049*	
C14	0.4488 (3)	0.3405 (3)	0.3818 (2)	0.0339 (13)	
C19	0.3959 (3)	0.3451 (3)	0.4193 (2)	0.0471 (16)	
H19	0.4122	0.3741	0.4552	0.057*	
C17	0.2956 (4)	0.2656 (4)	0.3514 (3)	0.0572 (18)	
H17	0.2434	0.2404	0.3405	0.069*	
C18	0.3194 (3)	0.3069 (4)	0.4038 (3)	0.0521 (17)	
H18	0.2834	0.3093	0.4294	0.063*	
C16	0.3476 (4)	0.2612 (3)	0.3151 (3)	0.0507 (17)	
H16	0.3309	0.2316	0.2796	0.061*	
C2	0.5837 (3)	0.4902 (3)	0.3689 (2)	0.0341 (13)	
P1	0.54959 (10)	0.39503 (10)	0.39594 (7)	0.0340 (4)	
C33	0.2450 (4)	0.1274 (4)	0.4703 (3)	0.069 (2)	
H33	0.2013	0.1138	0.4464	0.083*	
C35	0.3655 (6)	0.1416 (6)	0.4787 (4)	0.106 (3)	
H35	0.4063	0.1373	0.4622	0.127*	
C34	0.3056 (5)	0.1254 (5)	0.4453 (4)	0.096 (3)	
H34	0.3046	0.1126	0.4042	0.115*	
C36	0.3692 (5)	0.1647 (6)	0.5374 (4)	0.128 (4)	
H36	0.4139	0.1800	0.5603	0.153*	
C8	0.5630 (3)	0.3996 (3)	0.4755 (2)	0.0345 (13)	
C9	0.5823 (3)	0.4644 (3)	0.5082 (2)	0.0465 (16)	
H9	0.5902	0.5090	0.4882	0.056*	
C13	0.5536 (4)	0.3365 (4)	0.5059 (3)	0.083 (3)	
H13	0.5412	0.2913	0.4846	0.100*	
C12	0.5624 (5)	0.3390 (4)	0.5682 (3)	0.089 (3)	
H12	0.5555	0.2952	0.5889	0.107*	
C37	0.3063 (4)	0.1655 (5)	0.5630 (3)	0.093 (3)	
H37	0.3068	0.1781	0.6040	0.112*	
C11	0.5805 (4)	0.4029 (4)	0.5991 (3)	0.060 (2)	
H11	0.5862	0.4040	0.6414	0.072*	
C10	0.5904 (4)	0.4652 (4)	0.5695 (2)	0.0555 (17)	
H10	0.6031	0.5102	0.5912	0.067*	
C29	0.1603 (7)	0.3662 (5)	0.5344 (3)	0.089 (3)	
H29	0.1592	0.4122	0.5304	0.107*	
C30	0.2286 (5)	0.3681 (4)	0.5475 (3)	0.072 (2)	
H30	0.2739	0.4157	0.5522	0.086*	
O1	0.5906 (3)	0.3592 (3)	0.36468 (18)	0.0356 (12)	
O2	0.0922 (3)	0.0804 (3)	0.52650 (18)	0.0436 (13)	
N1	0.5587 (3)	0.2656 (4)	0.2408 (2)	0.0473 (17)	
C1	0.5031 (4)	0.2346 (4)	0.2139 (2)	0.0405 (16)	
S1	0.42411 (10)	0.18996 (11)	0.17744 (7)	0.0675 (6)	
N2	0.0419 (7)	0.1088 (7)	0.4014 (5)	0.059 (4)	0.667
C38	0.0671 (12)	0.1696 (9)	0.3841 (9)	0.065 (3)	0.667
S2	0.0997 (3)	0.25559 (18)	0.36159 (19)	0.0701 (11)	0.667
O3	0.0028 (11)	0.1221 (9)	0.4189 (6)	0.086 (5)	0.333
O4	0.0805 (12)	0.1092 (11)	0.3900 (8)	0.086 (5)	0.333
O5	0.0886 (13)	0.2223 (11)	0.3588 (10)	0.065 (3)	0.333
N3	0.0614 (15)	0.1602 (12)	0.3857 (12)	0.065 (3)	0.333

### Atomic displacement parameters (Å^2^)

**Table:**

	*U*^11^	*U*^22^	*U*^33^	*U*^12^	*U*^13^	*U*^23^
Eu1	0.02793 (16)	0.02793 (16)	0.0244 (3)	0.01396 (8)	0.000	0.000
Eu2	0.0534 (2)	0.0534 (2)	0.0211 (3)	0.02671 (11)	0.000	0.000
P2	0.0414 (10)	0.0455 (11)	0.0281 (8)	0.0197 (9)	0.0038 (7)	0.0029 (7)
C20	0.026 (3)	0.038 (3)	0.026 (3)	0.010 (3)	0.000 (2)	0.005 (2)
C26	0.052 (4)	0.050 (4)	0.025 (3)	0.026 (4)	0.005 (3)	0.007 (3)
C27	0.076 (5)	0.079 (5)	0.034 (3)	0.055 (4)	0.009 (3)	0.002 (3)
C25	0.057 (4)	0.032 (3)	0.031 (3)	0.015 (3)	0.004 (3)	0.006 (3)
C23	0.052 (4)	0.052 (4)	0.034 (3)	0.023 (4)	0.002 (3)	0.013 (3)
C21	0.048 (4)	0.037 (4)	0.034 (3)	0.017 (3)	0.002 (3)	0.000 (3)
C5	0.090 (6)	0.043 (4)	0.057 (4)	0.034 (5)	−0.002 (4)	0.009 (3)
C7	0.048 (4)	0.046 (4)	0.036 (3)	0.020 (3)	0.003 (3)	−0.010 (3)
C22	0.053 (4)	0.040 (4)	0.050 (4)	0.021 (3)	0.006 (3)	0.014 (3)
C24	0.059 (4)	0.047 (4)	0.038 (3)	0.014 (4)	−0.009 (3)	−0.005 (3)
C3	0.050 (4)	0.053 (4)	0.056 (4)	0.031 (4)	0.018 (3)	0.015 (3)
C31	0.069 (5)	0.056 (5)	0.035 (3)	0.029 (4)	0.004 (3)	0.003 (3)
C4	0.082 (6)	0.059 (5)	0.081 (5)	0.047 (5)	0.013 (4)	0.015 (4)
C32	0.042 (4)	0.057 (4)	0.042 (3)	0.028 (4)	0.009 (3)	0.014 (3)
C28	0.120 (8)	0.097 (7)	0.053 (5)	0.087 (7)	0.004 (5)	0.004 (5)
C6	0.052 (4)	0.040 (4)	0.048 (4)	0.000 (4)	0.013 (3)	0.000 (3)
C15	0.037 (4)	0.042 (4)	0.042 (3)	0.018 (3)	0.002 (3)	−0.008 (3)
C14	0.033 (3)	0.033 (3)	0.036 (3)	0.017 (3)	0.008 (2)	0.005 (2)
C19	0.045 (4)	0.058 (4)	0.040 (3)	0.027 (4)	0.010 (3)	−0.001 (3)
C17	0.036 (4)	0.060 (5)	0.078 (5)	0.026 (4)	−0.010 (3)	−0.010 (4)
C18	0.037 (4)	0.060 (5)	0.069 (5)	0.031 (4)	0.015 (3)	0.005 (4)
C16	0.055 (5)	0.045 (4)	0.052 (4)	0.025 (4)	−0.014 (3)	−0.020 (3)
C2	0.042 (4)	0.041 (4)	0.025 (3)	0.025 (3)	−0.003 (2)	−0.008 (2)
P1	0.0356 (10)	0.0403 (10)	0.0302 (9)	0.0221 (9)	−0.0010 (7)	−0.0040 (7)
C33	0.076 (6)	0.105 (6)	0.054 (4)	0.066 (5)	0.014 (4)	0.007 (4)
C35	0.121 (9)	0.176 (10)	0.073 (6)	0.114 (8)	0.057 (6)	0.039 (6)
C34	0.119 (8)	0.148 (9)	0.062 (5)	0.099 (8)	0.035 (5)	0.024 (5)
C36	0.064 (6)	0.236 (12)	0.101 (7)	0.089 (8)	0.013 (5)	0.030 (7)
C8	0.036 (3)	0.043 (4)	0.033 (3)	0.026 (3)	0.002 (2)	−0.003 (3)
C9	0.060 (4)	0.057 (4)	0.032 (3)	0.037 (4)	−0.003 (3)	−0.002 (3)
C13	0.161 (9)	0.067 (5)	0.044 (4)	0.074 (6)	−0.008 (4)	0.001 (4)
C12	0.160 (9)	0.090 (7)	0.049 (4)	0.086 (7)	−0.003 (5)	0.015 (4)
C37	0.066 (6)	0.156 (9)	0.060 (5)	0.058 (6)	0.026 (4)	0.015 (5)
C11	0.065 (5)	0.104 (6)	0.030 (3)	0.057 (5)	0.001 (3)	−0.004 (4)
C10	0.063 (5)	0.065 (5)	0.040 (3)	0.032 (4)	−0.001 (3)	−0.009 (3)
C29	0.176 (11)	0.087 (7)	0.037 (4)	0.090 (8)	0.018 (6)	0.011 (5)
C30	0.111 (7)	0.046 (5)	0.036 (4)	0.022 (5)	0.010 (4)	0.002 (3)
O1	0.036 (3)	0.043 (3)	0.035 (3)	0.026 (2)	−0.002 (2)	−0.009 (2)
O2	0.039 (3)	0.053 (3)	0.030 (2)	0.016 (3)	−0.0020 (19)	−0.003 (2)
N1	0.034 (4)	0.064 (4)	0.034 (3)	0.018 (3)	−0.013 (3)	−0.006 (3)
C1	0.054 (5)	0.057 (5)	0.028 (3)	0.041 (4)	0.009 (3)	0.002 (3)
S1	0.0499 (12)	0.0825 (15)	0.0536 (10)	0.0207 (11)	−0.0238 (8)	−0.0100 (9)
N2	0.063 (10)	0.086 (11)	0.038 (7)	0.045 (9)	0.017 (6)	0.001 (6)
C38	0.065 (6)	0.085 (8)	0.047 (5)	0.040 (7)	0.013 (4)	0.004 (5)
S2	0.096 (3)	0.049 (2)	0.067 (2)	0.038 (2)	0.0164 (17)	0.0274 (19)
O3	0.137 (15)	0.069 (8)	0.054 (8)	0.053 (10)	0.013 (8)	0.027 (6)
O4	0.137 (15)	0.069 (8)	0.054 (8)	0.053 (10)	0.013 (8)	0.027 (6)
O5	0.065 (6)	0.085 (8)	0.047 (5)	0.040 (7)	0.013 (4)	0.004 (5)
N3	0.065 (6)	0.085 (8)	0.047 (5)	0.040 (7)	0.013 (4)	0.004 (5)

### Geometric parameters (Å, º)

**Table:**

Eu1—O1^i^	2.292 (4)	C32—C37	1.350 (8)
Eu1—O1^ii^	2.292 (4)	C28—H28	0.9500
Eu1—O1	2.292 (4)	C28—C29	1.356 (11)
Eu1—N1^i^	2.397 (6)	C6—H6	0.9500
Eu1—N1	2.397 (6)	C15—H15	0.9500
Eu1—N1^ii^	2.397 (6)	C15—C14	1.368 (7)
Eu2—O2	2.277 (4)	C15—C16	1.400 (7)
Eu2—O2^iii^	2.277 (4)	C14—C19	1.403 (7)
Eu2—O2^iv^	2.277 (4)	C14—P1	1.804 (5)
Eu2—N2^iii^	2.359 (12)	C19—H19	0.9500
Eu2—N2	2.359 (12)	C19—C18	1.390 (7)
Eu2—N2^iv^	2.359 (12)	C17—H17	0.9500
Eu2—O3^iv^	2.637 (16)	C17—C18	1.379 (7)
Eu2—O3	2.637 (16)	C17—C16	1.369 (7)
Eu2—O3^iii^	2.637 (16)	C18—H18	0.9500
Eu2—O4^iv^	2.562 (17)	C16—H16	0.9500
Eu2—O4	2.562 (18)	C2—P1	1.800 (6)
Eu2—O4^iii^	2.562 (17)	P1—C8	1.791 (5)
P2—C20	1.799 (5)	P1—O1	1.522 (4)
P2—C26	1.803 (6)	C33—H33	0.9500
P2—C32	1.799 (6)	C33—C34	1.372 (8)
P2—O2	1.508 (5)	C35—H35	0.9500
C20—C25	1.387 (7)	C35—C34	1.321 (10)
C20—C21	1.381 (7)	C35—C36	1.381 (10)
C26—C27	1.390 (8)	C34—H34	0.9500
C26—C31	1.387 (8)	C36—H36	0.9500
C27—H27	0.9500	C36—C37	1.407 (9)
C27—C28	1.371 (9)	C8—C9	1.381 (7)
C25—H25	0.9500	C8—C13	1.377 (7)
C25—C24	1.391 (6)	C9—H9	0.9500
C23—H23	0.9500	C9—C10	1.376 (6)
C23—C22	1.375 (7)	C13—H13	0.9500
C23—C24	1.363 (7)	C13—C12	1.399 (7)
C21—H21	0.9500	C12—H12	0.9500
C21—C22	1.387 (6)	C12—C11	1.348 (8)
C5—H5	0.9500	C37—H37	0.9500
C5—C4	1.361 (8)	C11—H11	0.9500
C5—C6	1.369 (8)	C11—C10	1.353 (8)
C7—H7	0.9500	C10—H10	0.9500
C7—C6	1.376 (7)	C29—H29	0.9500
C7—C2	1.381 (7)	C29—C30	1.401 (11)
C22—H22	0.9500	C30—H30	0.9500
C24—H24	0.9500	N1—C1	1.150 (8)
C3—H3	0.9500	C1—S1	1.615 (7)
C3—C4	1.390 (8)	N2—C38	1.144 (14)
C3—C2	1.384 (7)	C38—S2	1.608 (13)
C31—H31	0.9500	O3—N3	1.283 (19)
C31—C30	1.393 (9)	O4—N3	1.280 (19)
C4—H4	0.9500	O5—N3	1.249 (16)
C32—C33	1.360 (7)		
			
O1^i^—Eu1—O1^ii^	89.68 (16)	C4—C5—C6	120.0 (6)
O1^i^—Eu1—O1	89.68 (16)	C6—C5—H5	120.0
O1^ii^—Eu1—O1	89.68 (16)	C6—C7—H7	119.7
O1^ii^—Eu1—N1^i^	95.6 (2)	C6—C7—C2	120.6 (6)
O1^i^—Eu1—N1^i^	87.14 (19)	C2—C7—H7	119.7
O1—Eu1—N1^i^	173.8 (2)	C23—C22—C21	118.5 (5)
O1^i^—Eu1—N1	95.6 (2)	C23—C22—H22	120.7
O1^ii^—Eu1—N1	173.8 (2)	C21—C22—H22	120.7
O1^i^—Eu1—N1^ii^	173.8 (2)	C25—C24—H24	119.9
O1—Eu1—N1	87.14 (19)	C23—C24—C25	120.2 (5)
O1—Eu1—N1^ii^	95.6 (2)	C23—C24—H24	119.9
O1^ii^—Eu1—N1^ii^	87.14 (19)	C4—C3—H3	120.2
N1^i^—Eu1—N1	87.9 (2)	C2—C3—H3	120.2
N1^i^—Eu1—N1^ii^	87.9 (2)	C2—C3—C4	119.7 (6)
N1—Eu1—N1^ii^	87.9 (2)	C26—C31—H31	120.7
O2—Eu2—O2^iii^	84.41 (16)	C26—C31—C30	118.6 (7)
O2—Eu2—O2^iv^	84.41 (16)	C30—C31—H31	120.7
O2^iii^—Eu2—O2^iv^	84.41 (16)	C5—C4—C3	120.6 (7)
O2^iii^—Eu2—N2^iii^	84.7 (3)	C5—C4—H4	119.7
O2—Eu2—N2^iii^	166.8 (3)	C3—C4—H4	119.7
O2^iv^—Eu2—N2^iii^	101.9 (3)	C33—C32—P2	118.4 (5)
O2—Eu2—N2	84.7 (3)	C37—C32—P2	122.1 (5)
O2^iii^—Eu2—N2	101.9 (3)	C37—C32—C33	119.3 (6)
O2—Eu2—N2^iv^	101.9 (3)	C27—C28—H28	119.9
O2^iv^—Eu2—N2	166.8 (3)	C29—C28—C27	120.1 (9)
O2^iv^—Eu2—N2^iv^	84.7 (3)	C29—C28—H28	119.9
O2^iii^—Eu2—N2^iv^	166.8 (3)	C5—C6—C7	120.1 (6)
O2^iii^—Eu2—O3^iv^	161.7 (4)	C5—C6—H6	119.9
O2^iv^—Eu2—O3^iv^	86.1 (4)	C7—C6—H6	119.9
O2—Eu2—O3^iv^	79.1 (4)	C14—C15—H15	120.1
O2^iv^—Eu2—O3	161.7 (3)	C14—C15—C16	119.7 (5)
O2^iii^—Eu2—O3	79.1 (4)	C16—C15—H15	120.1
O2—Eu2—O3	86.1 (4)	C15—C14—C19	119.7 (5)
O2^iv^—Eu2—O3^iii^	79.1 (4)	C15—C14—P1	118.1 (4)
O2—Eu2—O3^iii^	161.7 (3)	C19—C14—P1	121.8 (4)
O2^iii^—Eu2—O3^iii^	86.1 (4)	C14—C19—H19	120.2
O2^iii^—Eu2—O4	119.1 (4)	C18—C19—C14	119.6 (5)
O2—Eu2—O4	80.5 (5)	C18—C19—H19	120.2
O2^iv^—Eu2—O4	150.2 (5)	C18—C17—H17	120.2
O2^iv^—Eu2—O4^iii^	119.1 (4)	C16—C17—H17	120.2
O2^iii^—Eu2—O4^iii^	80.5 (5)	C16—C17—C18	119.6 (6)
O2^iv^—Eu2—O4^iv^	80.5 (5)	C19—C18—H18	119.8
O2—Eu2—O4^iii^	150.2 (5)	C17—C18—C19	120.4 (5)
O2^iii^—Eu2—O4^iv^	150.2 (5)	C17—C18—H18	119.8
O2—Eu2—O4^iv^	119.1 (4)	C15—C16—H16	119.6
N2—Eu2—N2^iv^	90.3 (4)	C17—C16—C15	120.9 (5)
N2^iii^—Eu2—N2^iv^	90.3 (4)	C17—C16—H16	119.6
N2^iii^—Eu2—N2	90.3 (4)	C7—C2—C3	119.0 (5)
N2—Eu2—O3^iv^	84.5 (5)	C7—C2—P1	119.1 (4)
N2^iii^—Eu2—O3^iv^	112.6 (5)	C3—C2—P1	121.9 (4)
N2—Eu2—O3	23.1 (4)	C2—P1—C14	108.1 (3)
N2^iv^—Eu2—O3	112.6 (5)	C8—P1—C14	107.6 (2)
N2^iv^—Eu2—O3^iv^	23.1 (4)	C8—P1—C2	108.0 (3)
N2^iii^—Eu2—O3	84.5 (5)	O1—P1—C14	110.5 (3)
N2^iii^—Eu2—O3^iii^	23.1 (4)	O1—P1—C2	110.5 (3)
N2^iv^—Eu2—O3^iii^	84.5 (5)	O1—P1—C8	111.9 (3)
N2—Eu2—O3^iii^	112.6 (5)	C32—C33—H33	119.0
N2^iii^—Eu2—O4^iii^	18.6 (5)	C32—C33—C34	122.0 (7)
N2^iv^—Eu2—O4^iv^	18.6 (5)	C34—C33—H33	119.0
N2^iv^—Eu2—O4	73.6 (5)	C34—C35—H35	119.8
N2—Eu2—O4	18.6 (5)	C34—C35—C36	120.4 (8)
N2^iv^—Eu2—O4^iii^	98.4 (5)	C36—C35—H35	119.8
N2—Eu2—O4^iv^	98.4 (5)	C33—C34—H34	120.3
N2^iii^—Eu2—O4^iv^	73.6 (5)	C35—C34—C33	119.5 (8)
N2—Eu2—O4^iii^	73.6 (5)	C35—C34—H34	120.3
N2^iii^—Eu2—O4	98.4 (5)	C35—C36—H36	120.2
O3^iii^—Eu2—O3^iv^	107.4 (4)	C35—C36—C37	119.6 (8)
O3—Eu2—O3^iv^	107.4 (4)	C37—C36—H36	120.2
O3^iii^—Eu2—O3	107.4 (4)	C9—C8—P1	122.5 (4)
O4^iv^—Eu2—O3	117.8 (5)	C13—C8—P1	119.6 (4)
O4—Eu2—O3^iii^	117.8 (5)	C13—C8—C9	117.9 (5)
O4—Eu2—O3	41.5 (5)	C8—C9—H9	119.6
O4^iii^—Eu2—O3	66.0 (6)	C10—C9—C8	120.9 (5)
O4^iv^—Eu2—O3^iii^	66.0 (6)	C10—C9—H9	119.6
O4^iv^—Eu2—O3^iv^	41.5 (5)	C8—C13—H13	120.0
O4—Eu2—O3^iv^	66.0 (6)	C8—C13—C12	120.0 (6)
O4^iii^—Eu2—O3^iii^	41.5 (5)	C12—C13—H13	120.0
O4^iii^—Eu2—O3^iv^	117.8 (5)	C13—C12—H12	119.6
O4^iii^—Eu2—O4	84.7 (7)	C11—C12—C13	120.9 (6)
O4—Eu2—O4^iv^	84.7 (7)	C11—C12—H12	119.6
O4^iii^—Eu2—O4^iv^	84.7 (7)	C32—C37—C36	118.9 (7)
C20—P2—C26	108.7 (2)	C32—C37—H37	120.5
C20—P2—C32	107.9 (3)	C36—C37—H37	120.5
C32—P2—C26	110.0 (3)	C12—C11—H11	120.3
O2—P2—C20	110.9 (3)	C12—C11—C10	119.5 (6)
O2—P2—C26	110.1 (3)	C10—C11—H11	120.3
O2—P2—C32	109.3 (3)	C9—C10—H10	119.5
C25—C20—P2	122.8 (4)	C11—C10—C9	120.9 (6)
C21—C20—P2	117.8 (4)	C11—C10—H10	119.5
C21—C20—C25	119.4 (4)	C28—C29—H29	120.2
C27—C26—P2	118.7 (5)	C28—C29—C30	119.7 (8)
C31—C26—P2	121.8 (5)	C30—C29—H29	120.2
C31—C26—C27	119.5 (6)	C31—C30—C29	120.8 (8)
C26—C27—H27	119.4	C31—C30—H30	119.6
C28—C27—C26	121.3 (7)	C29—C30—H30	119.6
C28—C27—H27	119.4	P1—O1—Eu1	165.8 (3)
C20—C25—H25	120.3	P2—O2—Eu2	168.6 (3)
C20—C25—C24	119.3 (5)	C1—N1—Eu1	173.5 (6)
C24—C25—H25	120.3	N1—C1—S1	178.8 (7)
C22—C23—H23	119.3	C38—N2—Eu2	164.6 (14)
C24—C23—H23	119.3	N2—C38—S2	177 (2)
C24—C23—C22	121.4 (5)	N3—O3—Eu2	110.8 (14)
C20—C21—H21	119.4	N3—O4—Eu2	115.3 (14)
C20—C21—C22	121.1 (5)	O4—N3—O3	92.0 (17)
C22—C21—H21	119.4	O5—N3—O3	134 (2)
C4—C5—H5	120.0	O5—N3—O4	134 (2)

Symmetry codes: (i) −*y*+1, *x*−*y*, *z*; (ii) −*x*+*y*+1, −*x*+1, *z*; (iii) −*y*, *x*−*y*, *z*; (iv) −*x*+*y*, −*x*, *z*.
